# Urinary Iodine Quantification in Epidemiological Studies: Optimization in Sample Preparation of the Sandell–Kolthoff Method

**DOI:** 10.1021/acsomega.5c08848

**Published:** 2026-05-27

**Authors:** Daniel Rosenkranz, David Vennen, Antje Kneuer, Gunnar Brandhorst, Nele Friedrich, Till Ittermann, Henry Völzke, Matthias Nauck, Martin Schlaud, Astrid Petersmann

**Affiliations:** 1 University Institute for Clinical Chemistry and Laboratory Medicine, Oldenburg 26133, Germany; 2 Department for Epidemiology and Health Monitoring, 9222 Robert Koch Institute, Berlin 13353, Germany; 3 University Institute for Clinical Chemistry and Laboratory Medicine, Greifswald 17475, Germany; 4 German Centre for Cardiovascular Research, Partner Site, Greifswald 17475,Germany; 5 Institute for Community Medicine, 60634 University Medicine Greifswald, Greifswald 17489,Germany

## Abstract

We present an optimized Sandell–Kolthoff (SK) spectrophotometric method for urinary iodine quantification, developed to enhance analytical accuracy, robustness, and suitability for high-throughput applications while minimizing reagent consumption. A stepwise refinement of an established protocol (Mod 1–4) was performed by adjusting reagent volumes, digestion temperature, and reaction conditions. The final optimized method (Mod 4) demonstrated excellent calibration linearity (*r* ^2^ = 0.99964) with a limit of quantification of 10.32 μg/L. Recovery rates for quality controls and certified reference materials ranged from 90 to 105%, with total imprecision (CV_total_) < 3% and analytical bias < 5%. Method comparison with inductively coupled plasma mass spectrometry (ICP-MS) and the Robert Koch Institute (RKI) reference method using Passing–Bablok regression and Bland–Altman analysis confirmed high concordance. Stability testing indicated that iodine concentrations remained unchanged during short-term storage at 4 °C, whereas repeated freeze–thaw cycles caused an average 16% decrease in measured concentrations. Surface contact with urine dipsticks introduced substantial exogenous iodine contamination, resulting in measurement overestimations of up to 45-fold. The optimized SK method provides a validated, precise, and resource-efficient approach for large-scale urinary iodine monitoring, aligning with World Health Organization (WHO) recommendations and addressing key limitations of existing colorimetric protocols.

## Graphical abstract

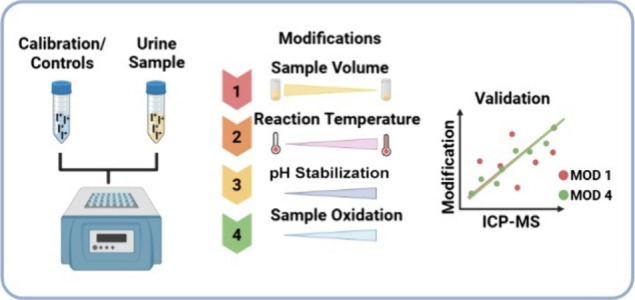

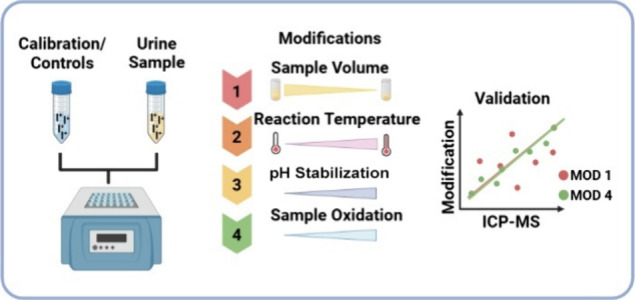

## Introduction

The trace element iodine plays a vital role in the human biology and is involved in the biosynthesis of thyroid hormones, including thyroxine (T4) and triiodothyronine (T3).^1^ These hormones regulate the metabolism and modulate the function of several tissues and body organs. They influence heart rate, energy consumption, weight management, blood sugar levels, cholesterol, mental health, muscle strength, digestion, and the growth and development of children.^2^ Iodine deficiency is associated with a spectrum of adverse health conditions, ranging from mild hyperthyroidism and goiter in adults to severe congenital disorders such as cretinism.^3^ In general, when dietary intake of iodine is insufficient, thyroid hormone production declines and the body attempts to compensate by enlarging the thyroid gland (goiter) to trap more iodine.

Given the broad implications of iodine on health, particularly in vulnerable populations such as pregnant women^4^ and infants,^5^ monitoring iodine status in populations is crucial for public health officials to implement and adjust iodine supplementation programs effectively.^6^ To assess the iodine status, urinary iodine concentration is widely recognized as a reliable indicator. This is because approximately 90% of ingested iodine is excreted in the urine, making it a convenient marker of dietary iodine intake.^7,8,3^ Urinary iodine measurement allows for the assessment of iodine status across populations and is instrumental in the monitoring of iodine deficiency disorders.

The WHO guideline for iodine deficiency and disorders contains thresholds and criteria for assessing iodine status based on median urinary iodine concentration (UIC) in a large population.^9^ For school-age children and adults, a median UIC of 100–199 μg/L indicates adequate iodine intake, while values below 100 μg/L indicate iodine deficiency (mild: 50–99 μg/L, moderate: 20–49 μg/L, severe: <20 μg/L). In pregnant women, the optimal range is 150–249 μg/L, with values below 150 μg/L indicating deficiency. Excess iodine intake is defined as ≥300 μg/L in the general population and ≥500 μg/L in pregnant women. Urinary iodine measurements should be conducted in a sufficiently large size of population-representative studies of 600–900 samples to ensure accurate population-level assessments.

To support such assessments, the WHO recommends the Sandell–Kolthoff method as a robust and widely applied colorimetric method for the assessment of iodine levels. Its simplicity and minimal technical requirements make it particularly suitable for implementation in resource-limited settings, including low- and middle-income countries, where access to advanced laboratory infrastructure and trained personnel is often restricted. The WHO outlines two protocols for sample digestion and subsequent iodine determination based on this method. In the preferred protocol, ammonium persulfate is used as the oxidizing agent, providing a safer and more practical alternative to perchloric acid, which is recommended as an optional oxidant. Although laboratories may implement additional modifications to digestion or measurement procedures to meet specific analytical requirements, such adaptations are not described in current WHO guidelines.^10^

In general, the Sandell–Kolthoff method involves the catalytic reduction of ceric ammonium sulfate (Ce^4+^) to cerium ammonium sulfate (Ce^3+^) by iodide (I^–^) in an acidic medium (eq 1).^10^

$${\mathrm{Ce}}^{4+}+{\mathrm{As}}^{3+}\overset{I^{-}}{\to }{\mathrm{Ce}}^{3+}+{\mathrm{As}}^{5+}$$ 1

In this redox system, iodide acts as a catalyst through a regeneration cycle involving iodine and arsenious acid. The catalytic sequence proceeds through three steps: (1) iodide reduces Ce^4+^ to Ce^3+^, forming iodine; (2) iodine is generated through oxidation of I^–^; and (3) iodine is subsequently reduced back to I^–^ by arsenious acid. The analytical signal arises from the decrease in absorbance at 420 nm as yellow Ce^4+^ is reduced to colorless Ce^3+^, and the reaction rate is proportional to the iodide concentration.

However, the traditional Sandell–Kolthoff method has notable drawbacks, including variability in sensitivity and specificity, depending on type of reagents which can influence the reactivity of cerium like ascorbic acid,^11^ metal ions (i.e., mercury, silver, iron, copper),^12^ high organic content, sulfur, sulfides or thiosulfates.^13^ Inductively Coupled Plasma Mass Spectrometry (ICP-MS) offers an alternative for iodine quantification and is renowned for its sensitivity, accuracy and capability of detecting trace elements at very low concentrations.^14^ However, the technique requires sophisticated infrastructure and technical expertise, making it expensive and thus less accessible for routine use in many parts of the world, particularly in resource-limited settings where public health burdens are often highest. Given these challenges, there is a compelling need to refine existing methodologies to get a balance between accessibility, cost, and analytical quality. The ideal method would be robust enough to comply with WHO standards for urinary iodine analysis, providing reliable data for public health decisions without the prohibitive costs and operational demands of techniques like ICP-MS.^15^

This study aimed to develop a robust and accurate method for urinary iodine determination by refining the Sandell–Kolthoff method. Based on established protocols from the Robert Koch Institute (RKI),^15^ systematic modifications were introduced to optimize chemical conditions—such as reactant concentrations and reaction temperature—to improve sensitivity and reduce variability. Each step was evaluated for accuracy, precision, and usability, and benchmarked against the gold standard ICP-MS. In addition, preanalytical steps like storage temperature and time, number of freeze–thaw cycles and possible contaminations from urine dip stick were tested as they play a vital role in conducting epidemiological studies that aim at accessing the iodine status of populations.

## Materials and Methods

### Method Optimization

For the establishment of the Sandell–Kolthoff method to determine iodine in urine in our laboratory, the method developed and established at the RKI serves as the basis.^16^ Initially, 500 μL of a urine sample were mixed with 900 μL of 1 mol/L ammonium persulfate (APS, Merck, Darmstadt, Germany, Art.-Nr. 1012019012) and 100 μL of 0.875 mol/L sodium hydroxide (NaOH, Pallets, Merck, Darmstadt, Germany, Art.-Nr. 1064980500), and digested in a heating block at 85 °C for 60 min. During this digestion step, ammonium persulfate serves as a strong oxidizing agent that breaks down organic matrix components and converts iodine from bound forms into reactive iodide, while NaOH enhances matrix solubilization and promotes the activation of persulfate. After digestion, the mixture is acidified to initiate the Sandell–Kolthoff reaction under the required acidic conditions.

The measurements of the digested solutions were performed on a Cobas Mira (Roche Diagnostics, Mannheim, Germany). For each analysis, 90 μL of the digested solution was automatically pipetted into a microvette. Subsequently, 200 μL of 0.0275 mol/L arsenic acid was added to the solution, which was then incubated for 150 s to facilitate the initial reaction. After incubation, 50 μL of 0.0378 mol/L cerium sulfate solution was added. The reduction of Ce^4+^ to Ce^3+^ was monitored after 600 s at a wavelength of 405 nm. The final quantification of the concentrations was done via a 9-point log–logit calibration in the range of 12.7–418.8 μg/L (Table 1, RKI).

**Table 1:** Overview of Parameters Used and Modified during Method Development with the Initial Method Used from RKI and the Recommended Method from WHO as Reference

method	RKI	Mod 1	Mod 2	Mod 3	Mod 4	WHO
Solutions						
sample (μL)	500	250	250	250	250	250
APS (μL) (1 mol/L)	900	450	450	1000	1000	1000
NaOH (μL) (0.875 mol/L)	100	50	50/100	100		
sample dilution factor	1:3	1:3	1:3	1:5.4	1:5	1:5
Digestion						
temperature (°C)	85	85	95	95	95	100
time (min)	60	60	60	60	60	60
Spectrometer						
method	endpoint	kinetic	kinetic	kinetic	kinetic	endpoint
calibration	log–logit4	FOK^a^	FOK^a^	FOK^a^	FOK^a^	linear/quadratic
Range (μg/L)						
lower	12.7	12.7	12.7	12.7	12.7	12.7
upper	418.8	418.8	571.1	571.1	571.1	418.8

a FOK, first order kinetic

To prepare the reagents for the Sandell–Kolthoff, 5.0 g of arsenic oxide (As_2_O_3_, certified by BAM, Merck, Darmstadt, Germany, U1–1019) was dissolved in 200 mL of 0.875 mol/L sodium hydroxide under stirring and gentle heating (max. 50 °C) and the addition of 300 mL of ultrapure water (Milli-Q, Merck Milipore, Darmstadt, Germany). Finally, 32 mL of concentrated sulfuric acid (96%, Suprapur, Merck, Darmstadt, Germany, Art.-Nr. 1007141000) and 25 g of sodium chloride (99.99%, Merck, Darmstadt, Germany, Art.-Nr. 1064060500) were added and filled up to 1000 mL with ultrapure water. For the 0.0378 mol/L ammonium cerium­(IV) sulfate solution, 24 g of ammonium cerium­(IV) sulfate dihydrate (Merck, Darmstadt, Germany, Art.-Nr. 383090-100G) were dissolved in 400–500 mL of 1.75 molar sulfuric acid and filled up to 1000 mL with ultrapure water. A calibration series in the range of 12.7–571.1 μg/L were prepared from a one molar potassium iodide (PanReac AppliChem, Darmstadt, Germany, Art.-Nr. 182256) standard solution.

Based on the method established by the RKI, several modifications were implemented (Mod 1–4). Instead of the Cobas Mira analyzer, a Gallery Analyzer (Thermo Fisher Scientific, Bremen, Germany) was used for photometric measurements. In contrast to the RKI method (end point measurement after 10 min) and the WHO protocol (method for measuring urinary iodine using ammonium persulfate, end point after 30 min), a continuous kinetic measurement approach was applied throughout Mod 1–4.

Immediately after the addition of the cerium­(IV) sulfate solution, the absorbance at 405 nm was recorded every 54 s over a total period of 10 min. Rather than using an end point measurement, the iodine concentration was determined based on the reaction rate under the assumption of first-order kinetics.^2^ The initial reaction rates were determined for all calibrators based on the linearized form of the absorbance–time curves. These rates were then plotted against the known iodine concentrations, and the resulting linear relationship was used to derive the apparent reaction rate constant, which reflects the system’s catalytic response to iodine. In contrast, the RKI method utilizes a log–logit calibration function for quantification, whereas the WHO protocol applies linear regression to end point absorbance values.

In the first modification step, sample and reagent volumes used in the reaction were reduced by half (Mod 1). This modification not only decreases the amount of sample material required but also aligns the protocol more closely with the procedure recommended by the WHO. Furthermore, it contributes to a reduction in chemical waste generation.

In a further step, the digestion temperature was increased from 85 to 95 °C in order to enhance the release of free, reactive iodine necessary for the Sandell–Kolthoff reaction (Mod 2). The volume of ammonium persulfate, serving as the oxidizing agent for the sample matrix, was increased in Mod 3 to 1000 μL to ensure more efficient matrix digestion. As a final modification (Mod 4), sodium hydroxide was omitted from the protocol due to concerns that it may interfere with the redox reaction kinetics, by altering iodine speciation and decreasing the chemical stability of arsenite. In Modifications 2, 3, and 4, the upper limit of the calibration range was extended, resulting in a new range of 12.7 to 571.1 μmol/L compared to the original range of 12.7 to 418.8 μmol/L.

Additional methodological details, including validation procedures, sample stability experiments, and contamination assessments, are available in the Supporting Information.

## Results

### Calibration

With each modification—adjusting reaction temperature, reagent proportions, and digestion conditions—the apparent reaction rate of the calibration curves increased by a factor of approximately 1.9 from Mod 1 to Mod 4 (−8.45 × 10^–4^ μmol/s to–4.48 × 10^–4^ μmol/s, Table 2).

**Table 2:** Calibration, Recovery, and Imprecision Data for Modifications 1–4^a^

		Mod 1	Mod 2	Mod 3	Mod 4
Calibration					
apparent reaction rate (μmol/s)		–8.45 × 10^–4^ (3.1 × 10^–5^)	–7.73 × 10^–4^ (2.8 × 10^–5^)	–4.14 × 10^–4^ (1.1 × 10^–5^)	–4.48 × 10^–4^ (9.7 × 10^–6^)
linearity (*r* ^2^)		0.99587 (0.00423)	0.99953 (0.00020)	0.99973 (0.00016)	0.99964 (0.00020)
Recovery (%)					
recipe L1		85.6 (6.7)	88.8 (3.5)	88.8 (11.5)	93.9 (3.4)
recipe L2		83.9 (2.3)	95.3 (2.8)	95.6 (2.6)	99.3 (2.2)
NIST L1			61.6 (31.9)	87.5 (11.2)	104.5 (0.9)
NIST L2			62.3 (29.2)	91.4 (11.0)	103.2 (1.3)
Imprecision					
recipe L1	CV_wd_ (%)	2.46	1.65	2.45	0.70
	CV_bd_ (%)	6.72	1.70	1.32	2.22
	CV_total_ (%)	7.13	2.33	2.75	2.31
recipe L2	CV_wd_ (%)	2.52	2.01	1.17	1.79
	CV_bd_ (%)	1.15	3.45	1.50	2.10
	CV_total_ (%)	2.79	3.96	1.95	2.60

a Calibration based on linearized first-order kinetics; *N* = 10 for Mod 1–2 and *N* = 8 for Mod 3–4. Recipe L1/L2 are lyophilized urine control samples (target values are 115 μg/L and 516 μg/L). *N* = 36 for Mod 1–2, *N* = 33/35 for Mod 3. NIST L1/L2 = SRM 3668 (target values 143 μg/L and 279 μg/L); *N* = 5. Parentheses indicate standard deviations. CV_wd_, within-day; CV_bd_, between-day.

Among all modifications, Mod 4 yielded the best apparent reaction rate with the smallest standard deviation (−4.48 × 10^–4^ μmol/s ± 9.7 × 10^–6^) and highest linearity (*r* ^2^ = 0.99964 ± 0.00020).

Recovery results, summarized in Table 2, showed a continuous improvement across method modifications. Mod 4 achieved the most consistent and accurate recoveries across all quality control levels, with 93.9% (±3.4%) for Recipe Level 1, 99.3% (±2.2%) for Level 2, 104.5% (±0.9%) for NIST Level 1, and 103.2% (±1.3%) for NIST Level 2. Compared to the other modifications, variability of recovery was reduced for the certified reference materials, particularly at NIST Level 1 with standard deviations of 31.9% in Mod 2 vs 0.9% in Mod 4 and also for NIST Level 2 (SD: 29.2% in Mod 2 vs 1.3% in Mod 4). Mod 2 showed the greatest inconsistency, between recoveries of different materials used: for the NIST levels, with mean recoveries below 65% and standard deviations exceeding 29% while SD of recovery for recipe controls were approximately 90% with SD between 2.8% and 3.5%. Mod 3 already demonstrated an improved recovery compared to Mod 2, with recovery rates for NIST materials rising above 87%. Variability of recovery for Mod 3was higher than in Mod 4, with standard deviations of 11.2% for NIST Level 1 and 11.0% for NIST Level 2. Due to the poor recovery observed for the Recipe controls, NIST SRM was not tested in Mod 1.

Imprecision data is given in Table 2. The largest improvement of imprecision was achieved by the improvements introduced in Mod 2. For Recipe Level 1, Mod 4 achieved a CV_wd_ of 0.70%, CV_bd_ of 2.22%, and CV_total_ of 2.31%, while for Level 2, CV_wd_ was 1.79%, CV_bd_ 2.10%, and CV_total_ 2.60%. By contrast, Mod 1 displayed the highest overall variation with CV_total_ of 7.13% for Level 1 and 2.79% for Level 2, mainly due to pronounced between-day variability (CV_bd_ = 6.72% for Level 1). Mod 2 and Mod 3 demonstrated intermediate imprecision, with Mod 3 yielding particularly low CV_total_ values at Level 2 (1.95%).

### Imprecision Profile

An imprecision profile was obtained for Mod 3 and 4, as they showed promising performance for potential routine application (Figure 1). At the lowest tested concentration (13 μg/L), Mod 3 showed a C*V*_total_ of 19.2%, while Mod 4 reached a value of 9.6%, corresponding to approximately half the imprecision. At the upper end of the calibration range, both methods showed low variability, with CVs of 4.0% (115 μg/L) and 2.4% (516 μg/L) for Mod 3 and 2.6% (115 μg/L) and 3.1% (516 μg/L) for Mod 4, respectively.

**Figure 1:**
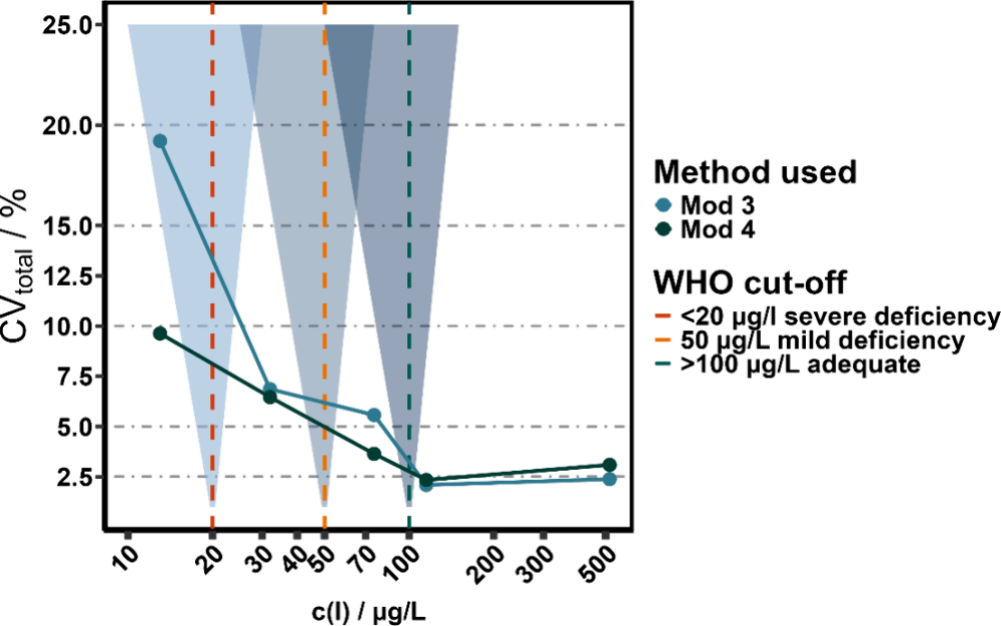
Concentration-dependent imprecision. For each batch, three replicates were analyzed for Recipe Control Level 1 (115 μg/L) and Level 2 (516 μg/L), and five replicates each for the in-house urine pool (75 μg/L) and potassium iodide solutions (13 and 32 μg/L). Total imprecision (CV_total_, %) of Modifications 3 and 4 was calculated from three independent batches across this concentration range. Triangles indicate the minimal detectable differences, defined as twice the standard deviation, at the WHO decision thresholds of 20, 50, and 100 μg/L.

### Analytical Limits

The assessment of analytical limits was restricted to Mod 3 and Mod 4 because these two modifications demonstrated substantially lower imprecision in the preceding experiments and therefore represented the only candidates suitable for a meaningful determination of LOB, LOD, and LOQ. In contrast, Mod 1 and Mod 2 exhibited markedly higher variability, which would have rendered their analytical limits unreliable and not representative of realistic routine performance.

Detection and quantification limits (Table 3) showed notable differences between Mod 3 and 4. The LOB is slightly higher for Mod 3 with 2.90 (0.84) μg/L compared to 2.53 (0.7) μg/L in Mod 4. The LOD calculated using the DIN 32645-based approach were 4.6 (1.26) μg/L for Mod 3 and 5.27 (1.52) μg/L for Mod 4. LOQ values were derived via both this DIN-based method and using CLSI EP17-A2 criteria. According to DIN 32645, Mod 3 showed an LOQ of 17.57 (5.08) μg/L and Mod 4 of 15.32 (4.22) μg/L. The LOQs obtained using CLSI EP17-A2 were slightly lower: 13.96 μg/L (CV 2.4%) for Mod 3 and 7.61 μg/L (CV 2.2%) for Mod 4.

**Table 3:** Detection and Quantification Limits for Modifications 3 and 4^c^

	Mod 3		Mod 4	
LOB (μg/L)	2.90 (0.84)		2.53 (0.7)	
LOD (μg/L)	5.27 (1.52)^a^		4.6 (1.26)^a^	
LOQ (μg/L)	17.57 (5.08)^a^	13.96 (2.4)^b^	15.32 (4.22)^a^	7.61 (2.2)^b^

a LOD and LOQ calculated according to DIN 32645.

b LOQ calculated according to CLSI EP17-A2.

c Values in parentheses represent standard deviations.

Representative spectrophotometric overlays, transformed FOK plots, and the calibration and blank data sets used for the analytical limits shown in Table 3 are provided in the Supporting Information.

### Method Comparison

The modified methods (Mod 2 to 4) were evaluated in comparison with the original RKI protocol and with ICP-MS, which served as the reference method. Linear regression analyses (Figure 2A–D) revealed strong correlations between all colorimetric methods and ICP-MS. Mod 3 and 4 showed the highest agreement with ICP-MS, with regression equations of *y* = 2.58 ± 5.61 μg/L + 0.91 ± 0.97*x* and *y* = 4.62 ± 6.95 μg/L + 0.89 ± 0.97*x*, respectively, and identical Spearman correlation coefficients (*r* ^2^ = 0.95). In contrast, Mod 2 showed a lower correlation (*r* ^2^ = 0.90) and a regression slope of 0.78 (*y* = −3.15 μg/L + 0.78*x*), indicating reduced proportional accuracy. The RKI method demonstrated reasonable alignment with ICP-MS (*y* = 5.86 μg/L + 0.84*x*, *r* ^2^ = 0.94).

**Figure 2:**
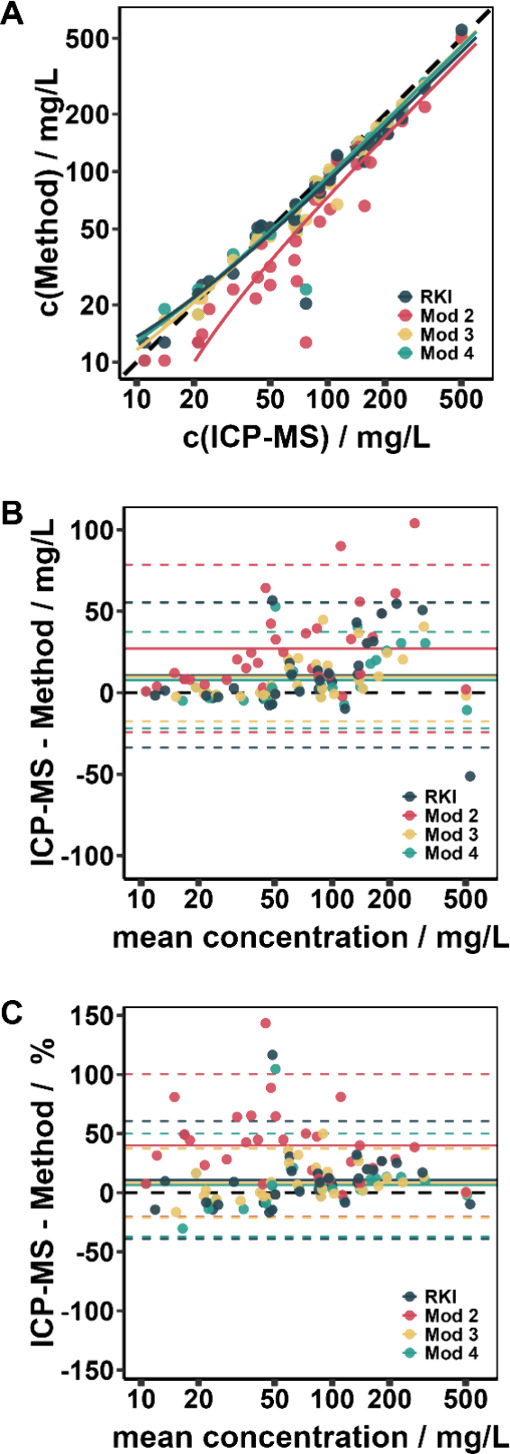
Method comparison of the Robert Koch Institute (RKI) protocol and three modified Sandell–Kolthoff methods (Mod 2–4) against ICP-MS for urinary iodine quantification. (A–C) Comparing the results of each colorimetric method with the ICP-MS reference using 31 urine samples. (A) Passing–Bablok regression analyses illustrating linear agreement, with dashed black lines. (B) Bland–Altman plots based on absolute differences and (C) relative (percentage) differences. Mean bias (solid line) and limits of agreement (dashed lines) are shown across the concentration range.

Bland–Altman analysis provided additional insight into the agreement with ICP-MS beyond correlation metrics. Mod 4 showed the smallest mean bias (4.13 μg/L) with relatively narrow limits of agreement (±35.21 μg/L), indicating that the method systematically overestimates ICP-MS results only slightly and maintains acceptable random variation across the concentration range. Mod 3 displayed a similarly low mean bias (5.82 μg/L) but even narrower limits of agreement (±25.48 μg/L), suggesting more consistent agreement, particularly at low and intermediate concentrations.

In contrast, Mod 2 demonstrated a pronounced positive bias (28.24 μg/L) and wide limits of agreement (±36.15 μg/L), pointing to systematic overestimation and reduced suitability for quantitative work. The RKI method also showed comparable deviation from ICP-MS (bias 7.32 μg/L; limits ± 36.3 μg/L) like Mod 3 and Mod 4 and a concentration-dependent trend, with larger discrepancies at elevated iodine levels.

Percentage differences showed a concentration-dependent pattern: Mod 3, Mod 4, and the RKI method tended to overestimate ICP-MS results below 100 μg/L, while a slight underestimation was observed at concentrations above this level.

In addition to its analytical performance, the optimized Sandell–Kolthoff protocol supports the parallel processing of up to 80 urine samples per batch, including calibrators and controls. With the current workflow, three batches can be prepared and measured within an 8-h working day, enabling a practical throughput of approximately 240 samples per day and demonstrating suitability for large epidemiological studies.

### Sample Stability and Contamination

#### Storage at 4 °C

The comparison of iodine concentrations between Day 0 and Day 5 is presented in Figure 3A. The median iodine concentrations remained stable between Day 0 and Day 5 with 37.05 μg/L. The corresponding interquartile range (IQR) were 40.86 μg/L and 48.73 μg/L, respectively, indicating similar dispersion. A Wilcoxon signed-rank test confirmed that the observed differences were not statistically significant (*p* = 0.293).

**Figure 3:**
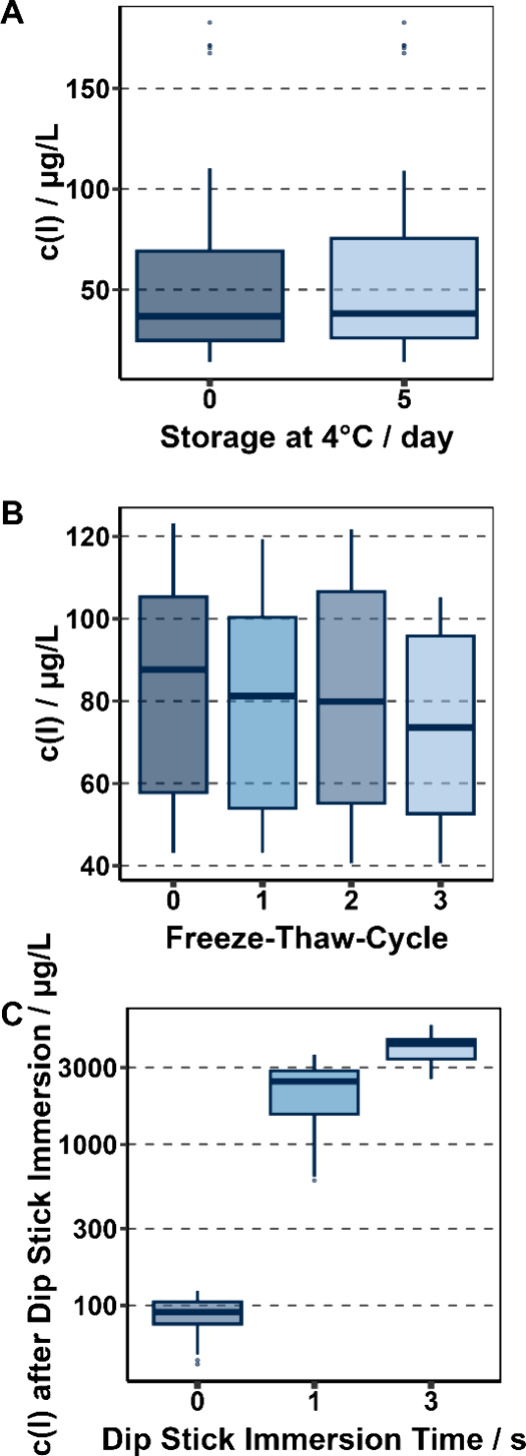
Impact of preanalytical conditions on urinary iodine measurements. (A) Iodine concentrations in 11 urine samples measured in quintuplicate before and after 5 days of storage at 4 °C (dark vs light blue). (B) Effect of 0–3 freeze–thaw cycles on nine samples (quintuplicate measurements; shading from dark to light blue). (C) Iodine concentrations in 10 samples exposed to dipstick contact for 0–3 s (triplicate measurements). All boxplots represent the full distribution of individual replicate measurements (A: *n* = 55; B: *n* = 45; C: *n* = 30), without prior averaging.

#### Freeze–Thaw Cycles

Iodine concentrations declined in all tested samples after three freeze–thaw cycles (Figure 3B). The median values showed a gradual downward trend with increasing freeze–thaw events: from 87.6 μg/L (0 cycles) to 81.2 μg/L (1 cycle), 79.9 μg/L (2 cycles), and 73.6 μg/L (3 cycles). The IQRs remained relatively consistent across groups, ranging from 43.15 μg/L to 51.40 μg/L, indicating no appreciable increase in variability. A Kruskal–Wallis test did not detect statistically significant differences between the groups (*p* = 0.493).

#### Dipstick Contamination

Iodine concentrations increased substantially with dipstick contact time (Figure 3C). In the control group without dipstick exposure, values ranged from 43.1 to 123.1 μg/L, with a mean of 88.8 μg/L. These concentrations reflect typical physiological levels and served as the reference for comparison. Following a one second exposure, iodine concentrations ranged from 596.5 to 3,616.5 μg/L, with a mean of 2,285 μg/L—corresponding to a 26-fold increase compared to the control mean. After three seconds of contact, concentrations increased further to a range of 2,544.5 to 5,539 μg/L averaging 4,005 μg/L, equivalent to a 45-fold elevation. The highest value observed was 5,539 μg/L.

## Discussion

### Analytical Improvements

The Sandell–Kolthoff reaction remains a widely used method for urinary iodine quantification due to its simplicity and cost-effectiveness.^10^ However, the approach has known limitations, including susceptibility to matrix effects and the need for manual end point detection.^11,12,17^ To address these shortcomings, the method was systematically refined through a series of modifications (Mod 1 to Mod 4) aimed at improving analytical precision, oxidation efficiency, and matrix tolerance.^18^

Mod 2 introduced a 10 °C increase in digestion temperature and a higher volume of sodium hydroxide. These changes were intended to promote a more consistent oxidation by adjusting the pH of the reaction mixture, which resulted in improved calibration linearity (*r* ^2^ > 0.9995). Recovery rates showed moderate improvements, particularly at lower iodine concentrations.

Mod 3 built upon this by increasing the concentration of APS, thereby enhancing oxidation efficiency and leading to more stable calibration curves, especially in the low-to-mid concentration range. The optimized digestion conditions also reduced matrix-induced variability and enabled more complete oxidation of both organic and inorganic iodine species, as shown by consistent recoveries across pooled urine, lyophilized controls, and certified reference materials (e.g., NIST SRM 3668).^11,12^

Mod 4 further optimized the protocol by omitting sodium hydroxide entirely and precisely adjusting APS concentrations. These changes improved redox kinetics and reduced the risk of ionic interference, supporting consistent oxidation regardless of iodine speciation. As a result, Mod 4 achieved intra- and interassay CVs ≤ 3%, aligning with international analytical quality standards.^9,19^

This level of precision surpasses the WHO threshold of 5% CV for reliable population-level iodine monitoring^9^ and compares favorably with other optimized S–K implementations. For instance, Haap et al. reported CVs between 2.5% and 5.5% using a microplate-based Sandell–Kolthoff method with high-temperature acid digestion (200 °C for 25 min) involving perchloric and nitric acid.^15^ Their approach enabled high throughput and good reproducibility but required specialized equipment and stringent safety conditions.

Similarly, Doggui et al. applied APS and sulfuric acid digestion in sealed tubes and achieved CVs of 2.1–4.0%, depending on sample matrix and storage stability.^18^ Their study emphasized the importance of preanalytical quality control, particularly regarding freeze–thaw stability and matrix-specific calibration.

Matrix-dependent differences in recovery were also evident in this study. Pooled urine and lyophilized controls, which predominantly contain organically bound iodine, showed higher recoveries and lower variation. In contrast, NIST SRM 3668—comprising more oxidation-resistant inorganic iodine species—consistently yielded lower recoveries, particularly under NaOH-containing conditions. These findings highlight the influence of iodine speciation and reagent chemistry on oxidation efficiency and analytical performance.^13^

To evaluate diagnostic performance across a clinically relevant concentration range, Modifications 3 and 4 were assessed between 12.7 and 516 μg/L. At the lower end (12.7 μg/L), near the WHO-defined threshold for severe iodine deficiency, Mod 3 yielded a CV_total_ of 19.2%, whereas Mod 4 achieved a markedly lower CV_total_ of 9.6%. At higher concentrations, both protocols showed high reproducibility (Mod 3:2.4%; Mod 4:3.1%).

In summary, the optimized Sandell–Kolthoff protocol—particularly Mod 4—demonstrates strong analytical performance, improved matrix tolerance, and practical applicability, making it well suited for routine diagnostics and large-scale iodine epidemiological monitoring in line with WHO recommendations. Beyond the stepwise refinements of digestion conditions, a key distinction from the classical WHO-recommended Sandell–Kolthoff procedure is the quantification strategy rather than in the underlying reagent chemistry. While the traditional assay relies on a single end-point measurement, the optimized protocol employs a first-order kinetic like evaluation in which all absorbance readings over time contribute to quantification. This kinetic approach yields concentration-dependent reaction rates that are less susceptible to short-term fluctuations, instrumental noise, and matrix-related effects. Consequently, the method achieves greater analytical robustness, allows shorter measurement times because full reaction completion is no longer required, and expands the linear calibration range—while remaining fully compatible with established SK workflows.

### Matrix Effects

The chemical form of iodine present in urine samples significantly influences the recovery behavior of analytical methods. In pooled human urine and lyophilized commercial controls (Recipe), iodine is primarily bound to organic compounds, which are generally more readily oxidized. In contrast, certified reference materials such as NIST SRM 3668 contain inorganic iodine species (e.g., iodide, iodate), which are more resistant to oxidation and thus pose a greater challenge for colorimetric methods unless properly adapted.^14,20^ A comparative evaluation of Mod 2 through 4, the RKI protocol, and ICP-MS revealed marked differences in analytical performance across these urinary iodine matrices. Although ICP-MS served as the analytical reference method, measurements in NIST SRM 3668 deviated by more than 10% from certified target values (Figure 4). This deviation is likely related to known matrix effects, particularly from inorganic iodine species, which can transiently impair plasma ionization or lead to minor carryover, even in otherwise robust systems.^14,21^ A systematic study by Landon et al.^21^ further emphasizes that, especially in complex urinary matrices, external calibration without matrix correction may introduce measurable bias. They observed signal suppression, memory effects, and drift under certain conditions. However, with appropriate calibration strategies and internal controls, ICP-MS retains its role as a high-precision method—provided matrix-specific validation is implemented.^21^

**Figure 4:**
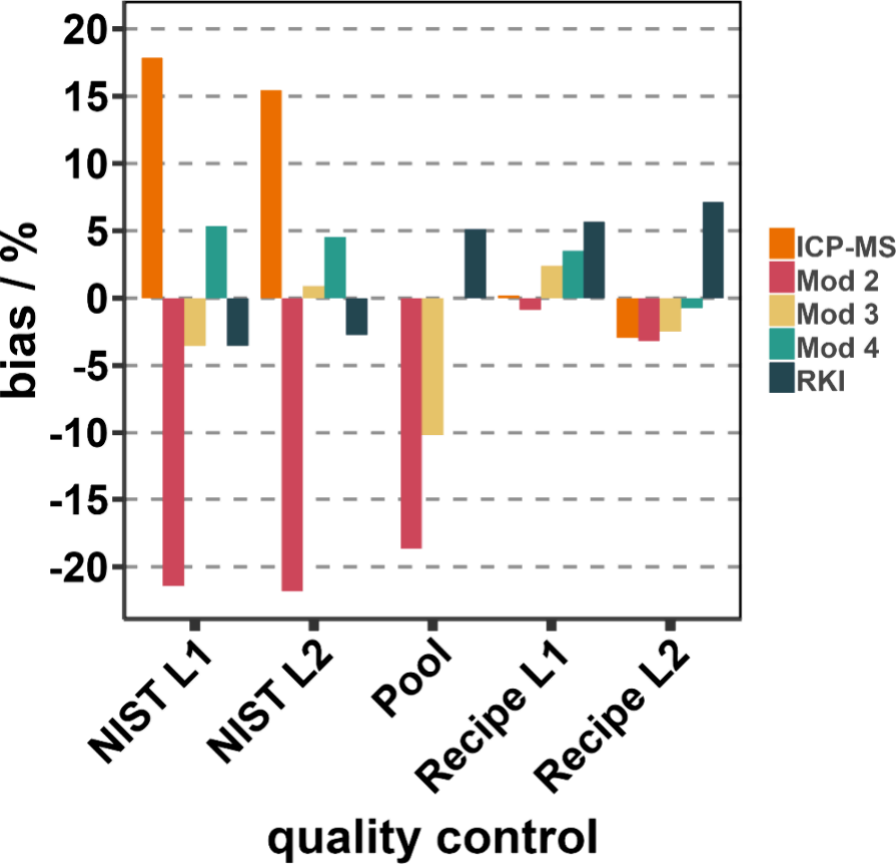
Quality assurance during method comparison. Five different quality control samples were measured for each participating laboratory and method used. These include NIST SRM 3668 Level 1 (L1) and Level 2 (L2), Recipe Level 1 (L1), and Level 2 (L2), as well as an in-house prepared urine pool.

Among the colorimetric methods tested, Mod 4 demonstrated the best overall agreement. In pooled urine and Recipe controls—both dominated by organically bound iodine—it maintained a bias within ±5% and yielded stable recoveries even in the NIST standards. Mod 3 and 4 performed adequately in all quality controls (urine pool, Recipe L1, L2 and NIST SRM 3668 L1, L2), while Mod 2 showed consistent underestimation of up to −20% in both NIST samples —likely attributable to incomplete oxidation of inorganic iodine species. The RKI protocol exhibited minor bias in lyophilized Recipe samples, indicating slightly reduced matrix compatibility in this context.

The superior matrix tolerance of Mod 4 can be attributed to its optimized ammonium persulfate concentration, the complete omission of sodium hydroxide to eliminate interfering side reactions, and improved redox kinetics. These adjustments likely enhance the oxidation efficiency for both organic and inorganic iodine species, as reflected in improved recovery performance. This is consistent with WHO recommendations, which emphasize the use of less hazardous, scalable, and analytically robust methods with sufficient sensitivity and matrix tolerance for population-level iodine monitoring.^10^

### Sample Stability and Interferences

The reliability of urinary iodine measurements depends critically on preanalytical handling, as iodine is known to be sensitive to degradation, volatilization, and contamination. Our data confirm that short-term refrigerated storage at 4 °C for up to 5 days preserves iodine stability. The median iodine concentration remained unchanged (37.05 μg/L), and a Wilcoxon signed-rank test revealed no statistically significant difference between Day 0 and Day 5 measurements (*p* = 0.293), in line with WHO recommendations for temporary sample storage.^9^ These findings support the use of refrigerated storage under field and laboratory conditions when immediate analysis is not feasible.

In contrast, repeated freeze–thaw cycles were associated with progressive iodine loss, as demonstrated in our experiments across four cycle groups. Median concentrations declined from 87.6 μg/L (0 cycles) to 73.6 μg/L (3 cycles), indicating a downward trend. However, variability remained stable across groups (IQR range: 43.15–51.40 μg/L), and the Kruskal–Wallis test showed no statistically significant differences (*p* = 0.493). Although these changes were not statistically conclusive, the trend supports previous evidence that each thawing step increases iodine volatility and may compromise measurement accuracy.^18^ Consequently, storage at −80 °C with a single thaw prior to analysis remains the recommended approach for long-term sample preservation.

While our data provide insight into short-term stability at 4 °C and into the effects of repeated freeze–thaw cycles, the present study does not address long-term storage stability at −20 °C or −80 °C. In many epidemiological and clinical studies, urine samples are archived for months or even years before iodine quantification, and storage practices vary considerably across laboratories. The long-term stability of urinary iodine under deep-freeze conditions therefore remains an important aspect for future investigation. This represents a limitation of the current study and underscores the need for systematic long-term stability assessments under real-world biobanking conditions.

A particularly critical and often underestimated source of analytical error is contamination by iodine-containing materials, most notably urine dipsticks. Our data demonstrated that even brief contact with dipsticks caused substantial artificial elevation of urinary iodine concentrations. This aligns with earlier reports of carryover contamination effects during iodine nutrition surveys.^22^ Since such contamination cannot be corrected retrospectively, strict avoidance of iodine-containing materials during sampling and preparation is essential. Nevertheless, we recognize that urine dipsticks are routinely used in many clinical workflows and may not always be avoidable. In such situations, we recommend implementing practical measures to reduce contamination risk, including minimizing the contact time between the dipstick and the urine sample, clearly documenting whether a dipstick was used before aliquoting, or collecting a fresh, dipstick-free aliquot for iodine determination. These steps can help mitigate or identify potential iodine overestimation introduced through dipstick exposure.

Overall, these findings underscore the need for rigorous adherence to standardized preanalytical protocols. Proper storage, minimal handling, and contamination control are key to ensuring the validity and comparability of urinary iodine data in research and public health surveillance.

## Conclusions

This study presents an optimized Sandell–Kolthoff method (Mod 4) for urinary iodine determination. Mod 4 achieved excellent calibration linearity (*r* ^2^ = 0.99964), near-complete recovery for controls and reference materials, and high precision (CV_total_ < 3%). As a validated, cost-effective alternative to ICP-MS, Mod 4 shows strong performance across iodine species, with broad matrix compatibility. It is well suited for large-scale and resource-limited monitoring. Compared to ICP-MS and the RKI method, Mod 4 demonstrated minimal bias and superior matrix tolerance, meeting WHO criteria for scalable, safe field methods. Its simplicity supports integration into population-level iodine monitoring. The study also stresses preanalytical integrity. Iodine remained stable for 5 days at 4 °C, but freeze–thaw cycles caused progressive loss, and dipstick contact led to overestimation. Rigorous sample handling and iodine-free materials are essential.

## Supplementary Material



## Notes

Supplementary figures, supplementary tables, detailed statistical results, model performance metrics, additional method descriptions, and supplementary figure captions (PDF)

## Notes

All authors consent to publication in ACS Omega.

## Notes

The TOC graphic was created entirely by one of the coauthors and is original, unpublished artwork. It complies with the ACS guidelines for TOC graphics.
