# Supplementary material for: Urinary Iodine Quantification in Epidemiological Studies: Optimization in Sample Preparation of the Sandell–Kolthoff Method
Source: ACS Omega. 2026 May 27;11(22):31940–8. doi: 10.1021/acsomega.5c08848 (PMC13261426; doi:10.1021/acsomega.5c08848)
Supplement: Supplementary file 1 [file ao5c08848_si_001.pdf]

Electronic Supplementary Information (ESI)

Urinary Iodine quantification in epidemiological studies:
Optimization in Sample Preparation of the Sandell-Kolthoff Method

Daniel Rosenkranz[1,*], David Vennen[1], Antje Kneuer[2], Gunnar Brandhorst[1], Nele Friedrich[3,4], Till Ittermann[5], Henry Völzke[4,5], Matthias Nauck[3,4], Martin Schlaud[2], Astrid Petersmann[1,3]

[1]University Institute for clinical chemistry and laboratory medicine, 26133 Oldenburg, Germany
[1]University Institute for clinical chemistry and laboratory medicine, 26133 Oldenburg, Germany
[2]Department for Epidemiology and Health Monitoring, Robert Koch Institute, Berlin, Germany
[3]University Institute for clinical chemistry and laboratory medicine, Greifswald, Germany
[4]German Centre for Cardiovascular Research, Partner Site Greifswald
[5]Institute for Community Medicine, University Medicine Greifswald, Germany

1. Validation

The validation of the method modifications was carried out in batch mode. Each batch included a complete 9-point calibration curve, commercial quality control samples (ClinChek, Recipe Chemicals and Instruments GmbH, Munich, Germany), a urine pool, and, where applicable, certified reference material (NIST SRM 3668, Gaithersburg, USA). All components were processed and measured together to minimize systematic variation between analytical runs. Ten calibration curves were performed for Mod 1 and 2, and eight for Mod 3 and 4, each measured in duplicate to verify reproducibility.

To assess measurement accuracy, within-day (CV_{wd}), between-day (CV_{bd}), and total variation (CV_{total}) were determined based on repeated measurements of Recipe controls at two

concentration levels (115 and 516 µg/L iodine). Per run at least two controls in different levels were performed (Mod 1 and Mod 2: n = 6 at 6 days, Mod 3: n = 10 - 12 at 3 days, Mod 4: n = 4 – 6 at 3 days)

Furthermore, recovery and analytical traceability were evaluated for Modifications 2 through 4 using the certified reference material NIST SRM 3668 (Level 1 = 142.7 µg/L, Level 2 = 279 µg/L), measured across five independent batches. The reference material was stored, handled, and used strictly according to the official NIST "Handling, Storage, and Use" instructions.

A detailed description of the kinetic data transformation, regression procedures, and calculation formulas for CV, recovery, LOB, LOD, and LOQ is included in the Supporting Information.

2. Imprecision profile

To evaluate the concentration-dependent coefficient of variation in relation to the minimal difference[1,2] of the WHO decision thresholds for iodine deficiency (20, 50, and 100 µg/L), an imprecision profile was generated specifically for Modifications 3 and 4. For this purpose, repeated measurements were conducted over three consecutive batches. This included for each batch, both Recipe control levels prepared and measured in triplicate, an in-house prepared urine pool (target value of 75 µg/L) of routine diagnostics left-over material five times, and potassium iodide solutions at concentrations of 0.1 (13 µg/L) and 0.25 µmol/L (32 µg/L) were each analyzed in five replicates. Finally, eight process blanks (analytical blank samples) were prepared and measured to identify and rule out any background signals or contamination introduced during the entire analytical procedure.

3. Analytical limits

The limits of detection (LOD) and quantification (LOQ) were established based on imprecision profile data derived from concentration-dependent variability experiments. LOQ values were determined using two complementary approaches: an exponential regression model fitted to the concentration-dependent imprecision data presented in Figure 1, and calculations based on the CLSI EP17-A2 guideline[3], where LOQ is defined as the lowest concentration with a CV ≤ 20%. Furthermore, LOD, and LOQ were calculated in accordance with DIN 32645[4] to evaluate the comparability of the two regulatory approaches. The simplified formulas 3×SD and 10×SD were applied for these estimates. The limit of blank (LOB) was calculated as mean_blank + 1.65×SD. Here, SD refers to the standard deviation derived from the blank replicate measurements. To ensure a statistically reliable estimation of the background signal (LOB) and its standard deviation (SD), eight blank measurements were performed.

4. Method comparison

To evaluate the performance of our modified methods (Mod 2–4), we compared them with both the established method of the Robert Koch Institute (RKI, Unit 22, Central Epidemiological Laboratory) and an ICP-MS method (Agilent 7900 in no gas mode) used as a reference by an accredited clinical diagnostics laboratory (Medical Laboratory Bremen, Bremen, Germany). For ICP-MS measurements the samples were diluted 1 to 10 in a 1 % nitric acid solution.[5]

A total of 31 urine samples were analyzed with each method. This sample set included 26 anonymized leftover urine samples from routine diagnostics, which were aliquoted into five portions of 1.5 mL each and stored at –80 °C until analysis. In addition, one pooled urine

sample, two commercial quality control materials (Recipe ClinChek Urine Control Level I and II), and two certified reference materials (NIST SRM 3668 Level 1 and Level 2) were included. All samples and controls were shipped to Bremen and Berlin on dry ice and anonymized to ensure blinded analysis and to prevent identification of control materials. Method agreement was assessed using Passing-Bablok regression[6] and Bland-Altman analysis[7].

5. Sample stability and contamination

Samples of large population-based studies are usually stored frozen. Therefore, stability of samples that underwent freeze-thaw-cycles was investigated. To assess the sample stability, two scenarios were tested. First, we tested whether the samples could be stored at 4 °C for up to five days before being re-measured (n = 11; concentration range: 15-104 µg/L, all samples prepared and measured five-times). This period reflects the typical conditions in clinical chemistry, where samples are often stored for several days at 4 °C in refrigerated archives in order to repeat measurements or perform new ones. Secondly, we evaluated how many times a sample could be frozen at -80 °C and thawed while maintaining consistent analytical quality (n = 9 with concentration ranging from 42 to 117 µg/L, all samples prepared and measured in triplicates).

Since iodine is used as a dye on urine dipsticks, we also investigated whether samples that underwent a dipstick analysis prior to iodine measurement are impaired by this treatment. [8] We evaluated the effect of dipstick (Combur 10, Roche Diagnostics, Mannheim, Germany) immersion on iodine measurements by applying different immersion times—no dipstick (control), 1 second (manufacturer's recommendation), and 3 seconds (prolonged contact)—to leftover urine samples (n = 10; iodine concentrations ranging from 43 to 123 µg/L, all samples

prepared and measured in triplicates). Iodine levels were then measured in both dipstick-treated and untreated samples to assess potential contamination related to contact duration.

Statistical comparisons between the respective groups were performed using the Wilcoxon rank-sum test and the Kruskal–Wallis test, as appropriate; to assess potential differences in iodine concentrations under the various storage and dipstick treatment conditions. P-values of 0.05 or lower were considered as statistically significant.

Formula used:

6. First-Order Kinetic (FOK) Evaluation of the Sandell–Kolthoff Reaction

The decrease in absorbance over time during the Sandell–Kolthoff reaction follows first-order kinetics with respect to triiodide concentration. The reaction progression can be described by:

$$A(t) = A_0 * exp(-k * t)$$

where

- A(t) = absorbance at time ttt,
- A_0 = initial absorbance,
- K = reaction rate constant (s^{-1}).

Taking the natural logarithm:

$$\ln\left(\frac{A}{A_0}\right) = -k * t$$

Thus, plotting ln(A/A_0) vs. time yields a straight line with slope = −k.

This kinetic parameter is used instead of a single endpoint to quantify iodide concentration.

The calibration curve therefore relates *k* to the known iodine concentration.

7. Spectrophotometric Overlays of FOK Measurements

7.1 Raw absorbance–time curves

In the raw data (Figure S1), absorbance decreases over time as Ce(IV) is reduced to Ce(III), with higher iodine concentrations producing a faster decline in absorbance. The separation of the two curves highlights the concentration-dependent reaction rate.

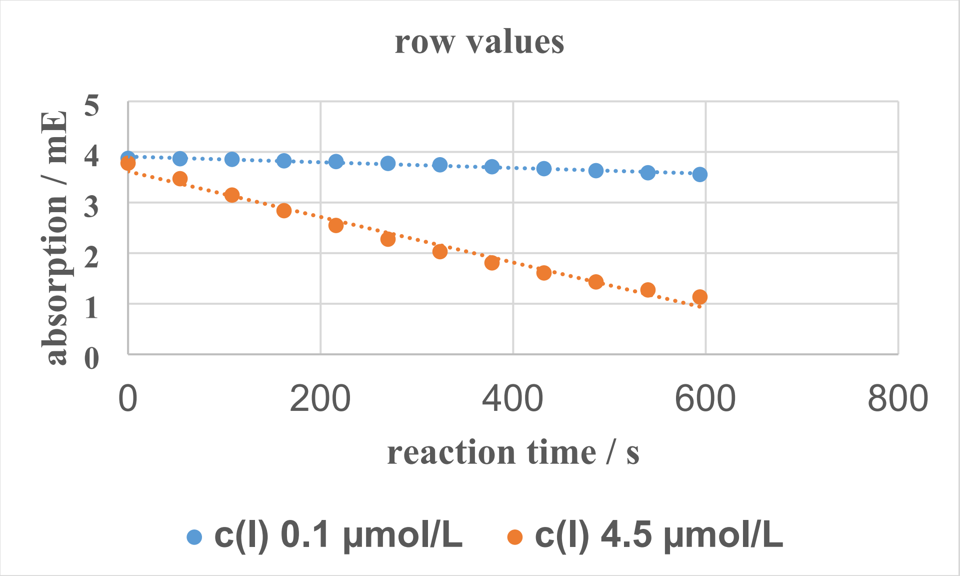

Figure S1: Absorbance vs. reaction time for two calibrants with iodine concentrations of 0.1 and 4.5 µmol/L.

7.2 Log-transformed kinetic plots

Figure S2 shows the corresponding transformed values, where absorbance data were normalized to the initial absorbance A_0 and evaluated according to:

$$\ln\left(\frac{A}{A_0}\right) = -k * t$$

After logarithmic transformation, the data yield straight lines whose slopes correspond to the negative reaction rate constant kkk. The linearity of the transformed curves confirms the validity of the first-order kinetic model across the investigated concentration range.

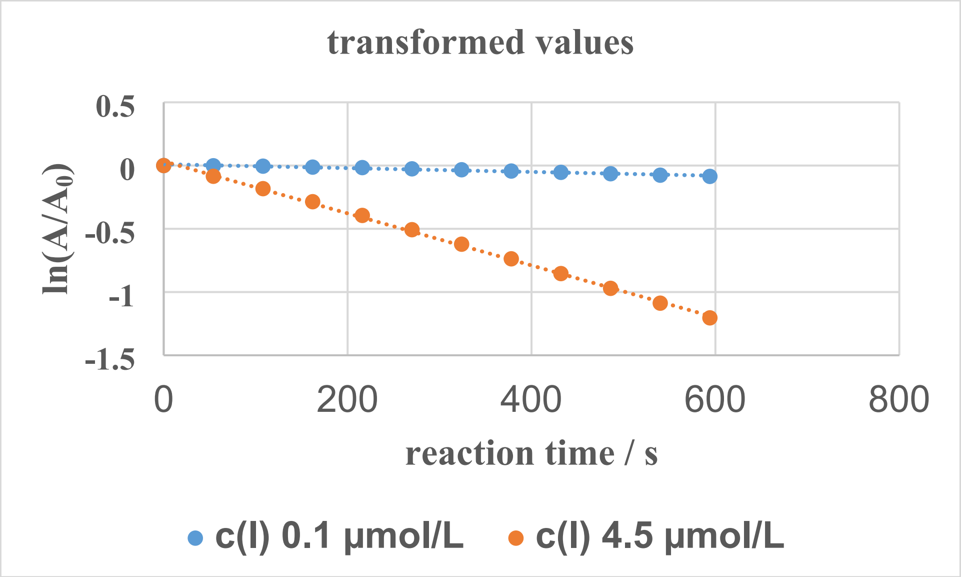

Figure S2: Log-normalized absorbance vs. reaction time two calibrants with iodine concentrations of 0.1 and 4.5 µmol/L.

8. Example Spectrophotometric Data for FOK Analysis

8.1 Raw endpoint and kinetic data (calibration series)

Table S1. Representative endpoint absorbances and calculated reaction rate constants.

Table S1: *Representative Calibration Data for the First-Order Kinetic (FOK) Evaluation of the Sandell–Kolthoff Reaction.*

Endpoint absorbances and corresponding first-order reaction rate constants (k) are shown for the calibration series used to generate the kinetic regression model.

	**Enpoint**	**Reaktion rate k**
**c(I) / µmol L^{-1}**	**Absorption / mE**	**k / µmol*s^{-1}**
12.69	3.56	-0.00018
63.45	3.245	-0.00035
101.52	2.97	-0.00049
126.9	2.83	-0.00056
190.35	2.50	-0.00080
253.8	2.18	-0.00103
304.56	1.96	-0.00121
418.77	1.56	-0.00160
571.05	1.11	-0.00217
**intercept**	3.43	-0.00013
**slope**	-0.56	-0.00045
**r^2**	0.9754	0.9998

8.2 Blank kinetics

Table S2. Blank endpoint absorbances and reaction rates.

Table S2. Blank Measurements Used for Estimation of LOB, LOD, and LOQ.

Endpoint absorbances and calculated reaction rate constants (k) for eight blank replicates

	**Enpoint**	**Reaction rate**
	**Absorption / mE**	**k /µmol*s^{-1}**
**Blank 1**	3.6106	-0.00015471
**Blank 2**	3.64348	-0.00013697
**Blank 3**	3.64668	-0.00015202
**Blank 4**	3.6411	-0.0001392
**Blank 5**	3.63016	-0.00013716
**Blank 6**	3.67837	-0.00013291
**Blank 7**	3.65814	-0.00014139
**Blank 8**	3.65873	-0.00014756

These values were used to compute the mean blank response and standard deviation (SD), which served as the basis for determining the limit of blank (LOB), limit of detection (LOD), and limit of quantification (LOQ).

9. Determination of LOB, LOD and LOQ

9.1 CLSI EP17-A2 Approach (Imprecision-Based LOQ Determination)

The CLSI-based determination of the limit of quantification (LOQ) follows the EP17-A2 guideline, which defines the LOQ as the lowest concentration that can be measured with a coefficient of variation (CV) of ≤ 20%.

To estimate the concentration corresponding to 20% CV, an imprecision profile is modeled, in which the CV is expressed as a function of analyte concentration. The following empirical regression model is applied:

$$CV(c) = a * c^{b}$$

This function represents a power-law regression.
Using the fitted parameters *a* and *b*, the LOQ is derived by solving the equation for the target CV:

$$c_{LOQ} = a * (CV_{target})^{b}$$

Example:

$$c_{LOQ} = 1.0214 * (20)^{b-0.891}$$

9.2 DIN 32645 approach (blank-based LOD/LOQ with calibration slope)

In contrast to the imprecision-based CLSI method, the DIN 32645:2008 framework determines analytical limits from:

1. The variability of blank measurements, and
2. The slope of the analytical calibration function.

Step 1 — Determine the limit of detection (LOD)

3. DIN defines the LOD via:

$$c_{LOD} = \frac{3 * SD_{Blank}}{b}$$

where b is the slope of the calibration curve.

Step 2 — Determine the limit of quantification (LOQ)

The LOQ is calculated analogously as:

$$c_{LOQ} = \frac{10 * SD_{Blank}}{b}$$

This approach therefore couples blank noise with analytical sensitivity (slope) and is independent of an imprecision profile.

10. Precision: Coefficient of Variation (CV)

For any set of repeated measurements:

$$CV/\% = \left(\frac{SD}{mean}\right) * 100$$

This applies to:

- within-day CV
- between-day CV
- total CV

as reported in the main manuscript.

11. Recovery

Recovery is calculated as:

$$Recovery/\% = \frac{measured\ value}{target\ value} * 100$$

This formula applies to:

- Recipe L1/L2
- NIST SRM 3668 L1/L2
- pooled urine QC materials

12. References

1. Ahmad-Nejad, P. *et al.* Revision of the "Guideline of the German Medical Association on Quality Assurance in Medical Laboratory Examinations – Rili-BAEK". *Journal of Laboratory Medicine* **48**, 263–306 (2024).
2. Keutmann, S. *et al.* Measurement Uncertainty Impacts Diagnosis of Diabetes Mellitus: Reliable Minimal Difference of Plasma Glucose Results. *Diabetes Ther* **11**, 293–303 (2020).
3. CLSI. Evaluation of Detection Capability for Clinical Laboratory Measurement Procedures; Approved Guidline - Second Edition (EP17-A2). Clinical and Laboratory Standard Institute; 2012.
4. DIN 32645:2008-11, 'Chemische Analytik - Nachweis-, Erfassungs und Bestimmungsgrenze unter Wiederholbedingungen - Bestimmung'.
5. Heitland, P. & Köster, H. D. Human biomonitoring of 73 elements in blood, serum, erythrocytes and urine. *Journal of Trace Elements in Medicine and Biology* **64**, 126706 (2021).

6. Passing, H. & Bablok, W. A New Biometrical Procedure for Testing the Equality of Measurements from Two Different Analytical Methods. Application of linear regression procedures for method comparison studies in Clinical Chemistry, Part I. *cclm* **21**, 709–720 (1983).
7. Bland, M. & Altman, D. G. Department of Clinical Epidemiology and Social Medicine, St George's Hospital Medical School, London SW17; and Division of Medical Statistics, MRC Clinical Research Centre, Northwick Park Hospital, Harrow, Middlesex.
8. Veyhe, A. S., Johannesen, H. L., Weihe, P. & Andersen, S. Urine test strips and iodine contamination: a tricky trick in iodine nutrition surveys. *Scandinavian Journal of Clinical and Laboratory Investigation* **82**, 251–256 (2022).
